# The “subluxation” issue: an analysis of chiropractic clinic websites

**DOI:** 10.1186/s40945-019-0064-5

**Published:** 2019-11-13

**Authors:** Alessandro R. Marcon, Blake Murdoch, Timothy Caulfield

**Affiliations:** Health Law Institute, Law Centre, University of Alberta, 91 University Campus, NW, Edmonton, Alberta T6G 2H5 Canada

**Keywords:** Chiropractic, Subluxation, Websites, Marketing, Regulation

## Abstract

**Background:**

Vertebral subluxation theories are controversial in chiropractic. Divisions are evident in the chiropractic community among those who align their practices to subluxation theories and those who do not. This study investigated how many clinics offering chiropractic in the Canadian province of Alberta promote a theory of subluxation, which health ailments or improvements were linked to subluxation, and whether the subluxation discourse was used to promote chiropractic for particular demographics.

**Methods:**

Using the search engine on the Canadian Chiropractic Associations’ website, we made a list of all clinics in Alberta. We then used Google searches to obtain a URL for each clinic with a website, totalling 324 URLs for 369 clinics. We then searched on each website for “subluxation” and performed content analysis on the related content.

**Results:**

One hundred twenty-one clinics’ websites (33%) presented a theory of vertebral subluxation. The health ailments and improvements discussed in relation to subluxation were wide-ranging. An observed trend was the marketing of chiropractic for children, which was observed on 29 clinic websites (8%).

**Conclusions:**

Based on the controversy surrounding vertebral subluxation, the substantial number of clinic websites aligning their practice with vertebral subluxation should cause concern for regulatory bodies.

## Background

The concept of vertebral subluxation – also called chiropractic subluxation or the vertebral subluxation complex – remains controversial, even within the chiropractic community [1–5]. The theory of vertebral subluxation, originally posited by D.D. Palmer at the turn of the century, has played a central role in the development of the practice of chiropractic [1, 5]. However, a considerable amount of research and commentary – often produced by the chiropractic community – has highlighted that there is no science to support the concept of vertebral subluxation [1–4, 6]. The concept is scientifically implausible, and there is no evidence to support the idea that it is associated with any ailment or the promotion of general health [1–5]. As such, in some jurisdictions, chiropractic organizations have taken a stand against vertebral subluxation. In 2010, for example, the General Chiropractic council in the UK stated, “The chiropractic vertebral subluxation complex is an historical concept but it remains a theoretical model. It is not supported by any clinical research evidence that would allow claims to be made that it is the cause of disease” [7]. Similarly, in 2015, various international bodies, including chiropractic schools, put forward a position statement that declared “the teaching of vertebral subluxation complex as a vitalistic construct that claims that it is the cause of disease is unsupported by evidence. Its inclusion in a modern chiropractic curriculum in anything other than an historical context is therefore inappropriate and unnecessary” [8]. The Alberta College and Association of Chiropractor’s 2008 position statement on the “Definition of Subluxation” makes no reference to vertebral subluxation but states that “the ACAC acknowledges and understands that the definition of a chiropractic subluxation may be ‘different from the current medical definition, in which subluxation is a significant structural displacement, and therefore visible on static imaging studies’” [9].

Indeed, divisions are evident among chiropractors over the role of subluxation and its related health problems [1, 5, 10]. The chiropractic community differs greatly in how subluxations are to be defined, and there currently exists no “gold standard” in the detection of subluxation [3, 11]. Furthermore, research has shown that in chiropractic education and training confusion exists among chiropractic students regarding the role subluxations play in chiropractic and whether these understandings of subluxation align with or contrast with an evidence-based approach [12, 13].

Despite the emerging consensus about the lack of science to support the subluxation theory, many chiropractors continue to embrace subluxation and offer services to patients based on its existence [1, 5]. The objective of this study was to examine the use of “subluxation” on the websites of Alberta health clinics offering chiropractic services. We examined how often the unscientific concept is promoted and used to market chiropractic services. We also sought to analyze the discourse surrounding vertebral subluxation, such as whether it was linked to specific medical conditions and used in marketing chiropractic services to particular demographics, including children.

## Methods

We used the Canadian Chiropractic Associations’ website [14] to obtain a list of health clinics offering chiropractic in the province of Alberta. On the 4th and 5th of January 2018, we used the “Find a chiro” search engine available on the website and recorded all clinics listed when searching for “Alberta”. This list totaled 493 clinics. We then searched for each of the 493 clinics using Google in order to match the clinic with a URL. In total we found a URL for 369 of the 493 clinics. These 369 clinics had a total of 324 URLs. This was because some clinics had multiple locations, or fell under an umbrella company, which shared the same URL. The two largest of these cases included one URL for 19 different clinics [15], and one URL for seven clinics [16].

We the inputted the formula: *subluxation site: [clinic URL]* into the Google Chrome search engine to capture all cases where “subluxation” appeared on the website. In cases where more than 10 page links appeared on a URL, we opened only the first 10 appearing on the search results. We then asked the following questions regarding the use of the “subluxation” content:

1. Is a theory of subluxation described (brought up and/or discussed in a health-related context)? If so, how is subluxation described?
2. Are health problems or general health improvements (use as a prevention or “wellness” strategy, increased energy, etc.) linked to “subluxation” (including: spine subluxation, vertebral subluxation, subluxation complex)?
3. Is “subluxation” theory critiqued (i.e. discredited, described as outdated or problematic, etc.)?
4. Is “subluxation” mentioned on the website in an alternative manner from that already coded?
5. Are any particular demographics focused on when discussing subluxation?

While coding, we observed some discourse that appeared to working towards creating anxiety or fear in (potential) clients. Due to its subjective nature, discourse of this sort was not formally quantified but some examples were captured and included in the results in order to highlight the presence of this problematic marketing.

To ensure validity, in all cases where “subluxation” appeared on a website, a screen shot of the discourse was taken with the clinic’s URL appearing in the URL bar at the top of the Google Chrome browser. In cases where subluxation appeared numerous times on the same page, we took a screen shot of each use. After our analysis, a researcher external to the project assessed all coding with respect to question #1. Any discrepancies were rechecked by the original coder.

## Results

### Theory of vertebral subluxation

Of the 369 clinics, 159 (43%) had websites containing the word “subluxation”, and 121 clinics’ websites (33%) present a theory of vertebral subluxation. That is, subluxation was described as a misalignment in the spine causing interference between the brain and body, and/or as having an impact on the nervous system or organ system functioning, and/or as affecting overall health or optimal health. Examples of these uses are displayed in Table 1. One additional website presented the vertebral subluxation theory but solely in the context of pets. Sixteen clinic websites (4%) mention “subluxation” in a variety of manners but do not clearly posit a theory of chiropractic vertebral subluxation.

**Table 1:** Ten text examples of clinic websites presenting a theory of chiropractic vertebral subluxation

Text related to theory, law, or philosophy or vertebral subluxations	
“Can Subluxations clear up by themselves? The chances of a subluxation healing itself is low because of the fast-paced lifestyle we life today. Dr. Battershill believes that once you have nerve interference created by a subluxation, that circuit breaker must be reset. This nerve interference affects the circuits of muscles, organs, glands, immune system and everything that is wired into that circuit, which is why it is so important to address subluxations as they come.” [17]	
“A subluxation is the result of spinal bones with improper motion or position affecting nerve communications between your brain and your body. A subluxation is a stress response. Muscles go into spasm. Spinal bones lock up. And adjacent nerves are choked or chafed. This interferes with the control and regulation of your body. This garbles communications between the brain and parts of your body. Distorted nerve communications can be an underlying cause of many health problems beyond headaches and back pain. For example, interference with nerve impulses going to or from your stomach: stomach problems. Your nervous system controls every cell, tissue, organ and system of your body. These nerve impulses travel through your spine. So having a spine free of subluxations is essential for optimal health. Only a chiropractic examination can detect subluxations. And only chiropractic adjustments can reduce their effect on your nervous system, naturally.” [18] (an additional 33 URLs have similar if not identical text under a heading “subluxation”. In some cases the word “subluxation” in the text appears as “vertebral subluxation”, and in one it appears as “nerve interference” [19])	
“When specifically treating the spine, chiropractors adjust vertebral subluxations. Which is a misalignment of one or more of the vertebrae in the spinal column, which causes alteration of nerve functions and interference to the transmission of mental impulses resulting in the lessening of the body’s innate ability to express its maximum health potential.” [20]	
“If a subluxation is present in the spine, a joint ceases to move properly, nerves become irritated interfering with nerve messages being sent, leading to organ and tissue malfunction. Subluxations also lead to decreased stimulation of the brain.” [21]	
“The nervous system controls and coordinates all functions of the human body. Subluxations/pinched nerves distort the nervous systems [sic] ability to control itself. That lack of proper function is what leads to disease. When you reduce/remove a subluxation you are allowing the body to physically function the way it was designed.” [22]	
“A subluxation is an incomplete or partial dislocation of a joint or organ. A spinal subluxation can impinge on a spinal nerve root causing symptoms in the areas served by those roots as the nerve is affected, the tissue or organ which is controlled by that nerve can become dysfunctional. A subluxation or an incomplete dyslocation should be assessed and needs a chiropractor for treatment.” [23]	
“I learned that a vertebral subluxation is a misalignment of any spinal vertebral segment and/or its articulation that causes interference of the nervous system which can over time negatively affected every cell, tissue, organ or organ system in the human body if not corrected.” [24]	
“Every function of your body is controlled by your central nervous system, and this function can be disrupted by misalignments [sic] in your spine. These are called subluxations. A subluxation creates interference in the function of your spinal nerves, and this can result in impaired functioning of your organs and endocrine system.” [25]	
“A vertebral subluxation alters blood chemistry via the hypothalamus. When a man or woman is subluxated, they have an increased release of stress hormones epinephrine and norepinephrine. These stress hormones, called catecholamines, release as a result of the “fight or flight” response. This response is a direct result of increased and sustained sympathetic nervous system tone. IT IS WELL KNOWN THAT INCREASED SYMPATHETIC TONE IS A MAJOR CAUSE OF CARDIOVASCULAR EVENTS AND DISEASE!” [26]	
“Chiropractic therapy is based on the hypothesis that reversible joint lesions of the spine produce far-ranging effects on the human body. Chiropractors rely on the spinal manipulation or (spinal adjustments) as their primary therapeutic tool of reversing the spinal dysfunctional units called the subluxation complex. Modern chiropractic does not claim to be a total therapy, but it does adhere to the idea that bio-mechanical dysfunction can have a profound effect not only on the musculoskeletal system but also other systems of the body.” [27]	

### Health problems or health improvements linked to subluxation

A wide range of health problems or general health improvements were linked to vertebral subluxation and are displayed in Table 2. It was not our objective to quantify the presence of each health topic. The health problems and improvements mentioned are extensive, including serious diseases and potential organ malfunction, a wide-range of child specific health problems such as ADHD, bed-wetting, and colic, and a range of other issues such as stomach and digestive problems, asthma, allergies, ear infections and blood pressure. In addition to the commonly expressed health issue of “nerve interference” (also called “nerve irritation” or “impaired nerve functioning”, etc.), other issues were raised such as “spinal decay” or “spinal degeneration”. It was also common for websites to state that uncorrected subluxations prevent a body from functioning “optimally”, and that a suboptimal body state will struggle to heal itself.

**Table 2:** Health problems and health improvements linked to chiropractic vertebral subluxation

Health problems or improvements:	
**Related to …** **Nerves** (functioning, interference, irritation, impinging); **Disease** (develop, onset or snowballing of, heart disease, prostate cancer, potential benefits for MS and Parkinson’s disease, hindering ability to cope with, “dis-ease”); **Neurology** (compromised integrity; altering supply); **Organ functioning** (influence on, decrease, digestion problems (constipation, diarrhea, bloating, gas), impaired reproductive organs, tense uterus impacting fetus’ growth); **Body “communication” and general functioning** (less or impaired signaling, less or impaired coordination, impaired function of endocrine system, dysfunctional brain, brain energy reduction, interference of innate intelligence, hindering abilities to adapt to stress, hindering rehabilitation and healing, tissue healing, hinder blood blow, blood pressure, disorders, fatigue, decreased vitality, dizziness (nausea), hormonal imbalances, overall immunity, impaired body temperature control, vision, hearing, asthma, hypertonicity); **Pain** (discomfort, mechanical stress, back (sciatica), chronic, leg, arm, muscle spasms, headaches); **Mobility** (spinal decay or spinal degeneration, wear and tear, decreased motion in spine and other areas, stiffness, balance, inflammation, decreased reaction time, decreased strength and endurance, damage bones, scoliosis, damaged facia); **Social and Other** (ADD/ADHD, autism, concentration, IQ, allergies, palpatory tenderness, ear infections, colds and flus, menstrual cramps, disinterest in life, sleeping difficulties, “failure to reach full potential”; impeding and enabling “optimal health”); **5 components of vertebral subluxation complex** (kinesiopathology, neuropathophysiology, myopathology, histopathology, pathophysiology); ---------------------------------------------------------------------------------------------- **Specifically for children:** nerve interference; interference in immune system; respiratory diseases; fever; earaches; ear infections; Sudden Infant Death Syndrome (SIDS); reduction in information to brain (brain flow); epilepsy; disrupting suckling response (for baby); disturbing optimal position vaginal delivery; colic; hyperactivity; “lowered resistance”; asthma; allergies; bed-wetting; “loss of focus in school”; constipation; colitis; “proper development”; growth and organ function; “better general well-being”; “sleep better”; “develop motor skills ahead of their peers”; “delayed development”; “poop consistently”; ADD/ADHD; immune system problems; learning disabilities; headaches; visual and hearing problems; “subluxations carry into adulthood and affect overall health and development”	

### Critiques of subluxation

There was only one website which outlined a somewhat critical perspective of subluxation. The text on this website, under the heading “Information for MDs” and the subheading “Education” read: “CMCC is Canada’s only English-speaking chiropractic college; it teaches an evidence-based medicine paradigm, as opposed to the traditional vertebral subluxation model sometimes taught in the United States” [28].

### Other mentions of “subluxation”

A total of 20 websites (5%) included the use “subluxation” in an alternative context. These included “subluxation” as the name of journal, a heading or subheading for a link, the name of a machine, the name of a process in a clinic (“the subluxation station”), or as part of a glossary.

### Particular demographics

A notable trend evident in the discourse was the marketing of subluxation for children. In total, there were 29 clinic websites (8%) marketing chiropractic for children (including newborns, infants, toddlers, etc.) when discussing subluxation. Table 2 presents the subluxation-related health issues for children, and Table 3 provides examples of the marketing for children.

**Table 3:** Text examples of clinic websites marketing chiropractic for children in the context of subluxation

Text showing the marketing of chiropractic for children in the context of subluxation	
“During the process of delivery, there is tremendous stress placed of the head and neck of the baby and often this stress causes Vertebral Subluxations of the very delicate spinal bones of the infant. This, in turn, affects the spinal cord and parts of the nervous system of the baby and can produce a myriad of health problems. These can appear seemingly unrelated to the process of delivery, and can affect your child months or years later. Subluxations associated with birth trauma could contribute to several ailments, such as colic, hyperactivity, ear infections, lowered resistance, asthma, bed-wetting, loss of focus in school, constipation, colitis, and a multitude of others. Research even suggests that spinal subluxations in the upper neck may be involved in Sudden Infant Death Syndrome (SIDS). According to German researcher G. Gutmann, MD, Ph.D., a “spinal check-up after birth should be obligatory.” His studies, spanning over a period of 35 years, concluded that over 80% of all children have a subluxation at birth.” [29]	
“Furthermore, the use of clamps and vacuums can easily lead to subluxations (spinal misalignment). Misalignments in the spine may be irritating your baby’s nervous system and causing the colic … Even a very gentle chiropractic adjustment can clear up major subluxations in an infant. Sometimes, it will take several treatments to restore the alignment of the spine and ensure healthy development is on track. A properly aligned spine restores the nervous system to its optimal function, allowing the body to heal and thrive. Many people have observed that even after a minor adjustment, their child’s capacity for breastfeeding is increased and they cry less because they are more comfortable. Once the child’s nervous system is fully functioning, they are at a decreased risk for allergies, ADD/ADHD, colds, flus, ear infections, asthma, and delayed development.” [30]	
“Newborns, infants and children get subluxated just like adults do. Often the first subluxations come from the birth process itself and that was most likely the start of the problems for this little guy. His birth was long and difficult and involved suction and forceps. Since the effects of subluxation can be seen where ever the nervous system goes (everywhere) newborns are our most important patients. Correcting subluxations in newborns allows innate intelligence to direct their proper development, growth AND organ function...It would have been best (and is always best) for this boy to have been checked by a Chiropractor as soon after birth as possible.” [31] On the same website: “I have never checked a child who was diagnosed with ADHD that did not have subluxation (interference with the spinal cord). When the subluxation is corrected these children decrease and most get off their Ritalin. Even now, among the medical community, there are warnings about the dangerous side effect of Ritalin. The problem is they are still looking for the solution to health problems in the form of a pill. Great health comes from within through an optimal spine and nerve system.” [32]	
“As the child begins to participate in regular childhood activities like skating or riding a bike, small yet significant spinal misalignments (subluxations) may occur. If neglected, the injuries during this period of rapid growth may lead to more serious problems later in life. Subtle trauma throughout childhood will affect the future development of the spine leading to impaired nervous system function. Any interference to the vital nerve system will adversely affect the body’s ability to function at its best.” [33]	
“In this video, Dr. Anrig explains how subluxations affect children. She explains the impact subluxations have on a child’s immune system as well as the impact on the nervous system and how that affects a child’s overall health.” [34]	
“Often times, misalignment or malfunction of the spine can occur sooner than we may think. Intrauterine constraint, abnormal positioning, and spinal distress from the delivery process can all lead to spinal subluxation (misalignment or malfunction). Everyday bumps, slips, and falls also have an effect on children’s developing spines and nervous systems. Ideally, your baby or child should have their spine and nervous system checked by a pediatric chiropractor as early as possible to promote proper function, especially after birth trauma or intervention. A tremendous amount of neurological development occurs during the infant and childhood years, so it’s important to ensure they have every opportunity to maximize nerve function during these critical periods. If left uncorrected, even minor subluxations and resulting nerve system dysfunction may develop into major health issues over time. Gentle and specific chiropractic adjustments can help to correct these subluxations, and allow for a properly functioning spine and nervous system at all ages. Many parents of children under regular chiropractic care have reported their children’s improvements in concerns such as recurring ear infections, colic, acid reflux, difficulty with feeding or attachment, torticollis, bedwetting, asthma, allergies, 2behavioural issues, neurodevelopmental disorders, immune function, and more. Chiropractic is not designed to, nor do we intend to treat any specific condition; chiropractors simply locate and correct interferences to the nervous system, so that the body is allowed to express health freely!” [35]	

### Fear-creating rhetoric in the subluxation discourse

There were three additional trends present in the subluxation discourse on the websites, which could be seen as an attempt to leverage fear. The first were numerous cases where subluxations were presented as asymptomatic (e.g. “Some subluxations may seem to cause no obvious symptoms”; “Pain and symptoms are a bad way to judge if you have subluxations or not”; “Individuals who are not currently experiencing pain or other discomfort are not necessarily ‘subluxation free’” etc.). The second included cases where subluxations were reportedly caused, or potentially caused by: concussions; whiplash (e.g. in car accidents); traumatic “physical causes” including “trauma to the body, repetitive motions affecting the spine, bad postural habits, improper workstation habits and design, and weak or imbalanced spinal musculature”; “Chemical causes” including “poor dietary and nutritional practices, drug and alcohol use and abuse, and the ingestion of chemical toxins in the foods we eat, air we breathe, and water we drink”; “emotional causes” such as stress or “stress management skills”, etc. These types of discourse were often coupled with the notion that only chiropractors are able to identify and correct subluxations and that regular checkups should take place. Lastly, the third trend were cases where subluxations were explicitly described as fatal, a “threat” or as “detrimental to health.” See Table 4 for examples:

**Table 4:** Fear-creating rhetoric on clinic websites

Fear-creating rhetoric	
“Symptoms poor judge of health – how do you know if you’re healthy? The first symptom of problems like heart disease, hypertension and cancer is often death! Does a loved one have pre-symptomatic subluxations?” [36]	
“However, the misalignment of the spine and pelvis have a far more crucial consequence to a mother and her developing baby. In chiropractic, these misalignments are called subluxations. These insidious blockages cause stress overload to the mother’s nervous system, potentially impairing any of her vital systems and organs. During pregnancy, when a mother’s body systems are basically functioning for two, any interference to the nerve transmission supporting their function can be detrimental to her health and well-being, as well as the growing baby’s.” [37]	
**“Subluxations ‘The Silent Killer’”** Subluxations can have devastating effects on your overall health and well-being.”; “Subluxations are a serious threat to your newborn’s health.” [38]	
“All people who have been in in an accident or trauma should see a chiropractor to have their spinal columns checked for nerve pressure caused by vertebral subluxations or spinal stress. A chiropractic adjustment can make the difference between life and death, between a life with pain, disability and sickness and a life of true recovery, activity and accomplishment.” [39]	

## Discussion

Our analysis shows that the marketing of chiropractic services on the basis of vertebral subluxation is undertaken by nearly a third of clinics in Alberta that have a working website. Considering that we could not find a URL for more than 120 websites, it is likely that additional clinics in the province align their practices with notions of vertebral subluxation. At least in Alberta, vertebral subluxation is adopted by a significant number of clinics. This is not a fringe issue. Rather, it appears central to the practice of a substantial number of clinics.

The health ailments and/or health improvements linked to the vertebral subluxation discourse on the websites are wide-ranging (see Table 2). While quantifying the presence of these subluxation-related health topics fell outside of the scope of this study, a lack of consistency across the clinics was observed. For example, some clinics associate subluxation with diseases like prostate cancer, MS or Parkinson’s disease, others with pregnancy and fetus development, and others with stomach issues, autism, ADD/ADHD or hormonal imbalances. Not all of the clinics’ websites include the marketing of chiropractic for newborns, infants and children, but some do.

Overall, this analysis provides insight into the degree to which chiropractic services are science-based, and raises interesting questions about regulation. Currently, chiropractic is a self-regulated profession in Canada. If the profession claims to be science-based, self-regulating chiropractic colleges should consider whether it is appropriate to discuss subluxation in an unscientific manner on their websites. Conversely, if these colleges have no issue with the kinds of subluxation-related discourse presented here, they should make official statements declaring this position – at which point legislators could consider what this means in the context of health policy and self-regulation. In the Alberta College and Association of Chiropractor’s 2008 position statement “Definition of Subluxation”, it reads, “The ACAC regulates the chiropractic profession in Alberta under the Health Professions Act. The ACAC is dedicated to regulating the chiropractic profession in a manner that supports quality care and upholds public trust; as well as furthering awareness and understanding of the benefits of chiropractic care” [9]. To what degree does the discourse presented here align or contrast with this statement? For example, Canadian paediatricians have raised concerns about the marketing of chiropractic to children [40], and The Canadian Pediatric Society states that “parents should be made aware that there is a lack of substantiated evidence for the theory of subluxated vertebrae as the causality for illness in children” [41]. This study identified 29 clinics marketing chiropractic for children solely in the context of subluxation discourse.

An extensive amount of research has highlighted the problematic marketing claims by chiropractic clinics from numerous perspectives [42, 43]. Some research has even raised the issue of appealing to fear in the context of chiropractic [44]. This study found rhetoric that would support those concerns, including discourse about potentially dangerous subluxations, which are potentially asymptomatic, and which may be caused by accidents, “poor dietary and nutritional practices”, “sitting”, “toxins” and/or “stress”. Furthermore, targeting parents with young children, in the process of having children, having recently been in an accident or suffering from a concussion, would certainly seem to be working contrary to the ACAC’s mandate of “regulating the chiropractic profession in a manner that supports quality care and upholds public trust” [39]. It is hoped that regulatory bodies will recognize these types of discourse as problematic and will act accordingly.

### Limitations

There were clinics in the province for which no URL could be found using the Google search engine. It is possible that the clinics without URLs present and/or align themselves to a perspective of vertebral subluxations differing from the trends we observed in our study. This study only examined the representations on clinic websites. We don’t know what information patients are provided at the relevant clinics. Also, this study identified 29 clinics marketing chiropractic for children solely in the context of subluxation discourse. It is possible that more clinics are marketing – and perhaps aggressively marketing – chiropractic for children.

## Conclusion

Based on the controversy surrounding vertebral subluxation, the substantial number of clinic websites in Alberta aligning their practice with vertebral subluxation should receive attention from relevant regulatory bodies. Corresponding regulatory bodies should act accordingly to determine which actions should be taken to clarify the numerous issues raised in this study.

## Data Availability

The datasets used and/or analysed during the current study are available from the corresponding author on reasonable request.
